# Highly Sensitive RNA-Based Electrochemical Aptasensor for the Determination of C-Reactive Protein Using Carbon Nanofiber-Chitosan Modified Screen-Printed Electrode

**DOI:** 10.3390/nano12030415

**Published:** 2022-01-27

**Authors:** Mahmoud Amouzadeh Tabrizi, Pablo Acedo

**Affiliations:** Electronic Technology Department, Universidad Carlos III de Madrid, 28911 Leganés, Spain

**Keywords:** aptasensor, electrochemical measurement, C-reactive protein, carbon nanofiber, screen printed electrode, redox indicator

## Abstract

C-reactive protein (CRP) is one of the biomarkers related to coronavirus disease 2019 (COVID-19). Therefore, it is crucial to develop a highly sensitive, selective, and cost-effective biosensor for the determination of CRP. In this study, we designed an electrochemical aptasensor. For this purpose, the surface of a carbon screen-printed electrode was first modified with a carbon nanofiber-chitosan (CNFs-CHIT) nanocomposite. After that, the amino-terminal RNA aptamer probes were linked to the amino groups of CHIT via glutaraldehyde as the cross-linker. Finally, methylene blue (MB) as a redox probe was self-assembled on the surface of the aptasensor. The obtained results indicated that the CNFs-CHIT nanocomposite increased the surface coverage of the aptamer up to 5.9 times. The square-wave voltammetry was used for the measurement of CRP concentration in the linear range of 1.0–150.0 pM. The obtained results indicated that the signal had a logarithmic relationship with the concentration of CRP. The limit of detection (LOD) was obtained to be 0.37 pM. The dissociation constant (K_d_) that demonstrates the affinity of the aptamer probe to its target was found to be 0.93 pM. The analytical performances of the proposed RNA aptasensor were better than the previously reported aptasensors for CRP. The proposed aptasensor was also applied for the determination of CRP in the human plasma samples. The obtained results indicated that there were no statistically significant differences between the responses of the proposed RNA aptasensor and an enzyme-linked immunosorbent assay kit (ELISA). The analytical performances of the proposed RNA aptasensor described in this paper are better than previously reported aptasensors for CRP determination.

## 1. Introduction

Sepsisis a potentially fatal response of the body to an infection that can lead to death in the framework of many diseases like coronavirus disease 2019 (COVID-19) [1]. The common method for recognizing sepsis is the measurement of the concentration of inflammation biomarkers in the human blood sample. C-reactive protein (CRP) is one of the common inflammation biomarkers. CRP levels higher than 10 ppm (86.8 nM) are considered a sign of serious infection [2]. Therefore, it is crucial to design highly sensitive, selective, and cost-effective sensors.

Aptasensors are made with a nucleic acid chain as bioreceptors [3] have been used for CRP measurement [4,5,6]. The aptasensors have several advantages compared to antibody-based biosensors (immunosensors) including: (1)They are smaller than antibodies (10-fold) [7] that help immobilize more of them on the surface of a transducer; (2) they can interact with redox indicators and self-assemble them on the surface of the transducer (like methylene blue, hexaammine ruthenium [8,9]); (3) they are modifiable with different functional groups like amine and thiol [3]; and (4) they are a low-cost bioreceptor. In the fabrication of an aptasensor, the aptamer is immobilized on the surface of a transducer. The transducer then is used to convert the interaction of the biomolecule/bio-recognizer (like a CRP/RNA aptamer) into a measurable signal [10,11]. Among the various transducers, the electrochemical transducers have several advantages in detecting the aptamer–biomarkers interaction such as their sensitivity, portability, accuracy, and rapid response time [12,13]. Jarczewska and her co-authors have presented an electrochemical RNA-based aptasensor to measure CRP concentration by self-assembling a thiolated RNA aptamer on the surface of a bare gold electrode [8]. They indicated that their proposed RNA aptasensor had a high affinity to the CRP (25.9 pM) and could be used for the measurement of CRP concentration up to 100.0 pM with a limit of detection of 1.0 pM. Their proposed electrochemical RNA-based aptasensor had a better analytical performance compared to the surface plasmon resonance (SPR)-based RNA aptasensors [14].

Nowadays, nanomaterials have been used for the fabrication of highly sensitive electrochemical biosensors [15,16,17,18,19]. Among the various nanomaterials, carbon nanofibers (CNFs) have several obvious advantages such as high surface area, low cost, easy fabrication, and biocompatibility [20]. To the best of our knowledge, the use of the CNFs and chitosan (CHIT) for the fabrication of the RNA-based aptasensor to detect CRP has not been reported yet. Both CNFs and CHIT are bio-friendly materials that provide a high degree of biocompatible microenvironment for the bio-recognizer, improving the stability of the biosensor [21,22,23]. 

In this research work, an electrochemical aptasensor was designed by using CNFs and CHIT for the determination of CRP. For this purpose, the amino-terminal RNA aptamer was immobilized on the surface of a carbon screen-printed electrode (CSPE) modified with CNFs and CHIT (CSPE/CNFs-CHIT) by using glutaraldehyde (GLU) as a cross-linker. Methylene blue (MB) as a redox indicator was then dropped on the surface of the electrode to interact with the immobilized RNA aptamer. According to the previous report, the positively charged MB can electrostatically interact with the negatively charged phosphate groups of the aptamer [24]. The obtained results indicated that the surface coverage of the immobilized aptamer in the proposed aptasensor (CSPE/CNFs-CHIT-RNA aptamer-MB) was higher than those previously reported for RNA-based [8] and DNA-based aptasensors [25] for CRP measurement. The proposed RNA aptasensor exhibited good sensitivity, affinity, stability, and reproducibility.

## 2. Materials and Methods

### 2.1. Reagents and Chemicals

MB, CHIT, potassium chloride (KCl), calcium chloride (CaCl_2_), sodium chloride (NaCl), sodium dihydrogen phosphate (NaH_2_PO_4_), Tris(hydroxymethyl)aminomethane (Tris), sodium acetate (NaAc), and GLU were purchased from Cymit Química (Barcelona, Spain). CNFs, bovine serum albumin (BSA), human serum albumin (HSA), human immunoglobulin G (HIgG), and CRP (~115 kDa) were purchased from Sigma Aldrich (Madrid, Spain). CRP-Human Enzyme-Linked Immunosorbent Assay (CRP-ELISA) was obtained from Thermo Fisher Scientific (Waltham, MA, USA). Carbon screen-printed electrodes (CSPE) were purchased from Metrohm-Drop Sens (Llanera, Spain). The amine terminal RNA aptamer probe (purified by HPLC) was purchased from Nzytech (Lisboa, Portugal). The RNA aptamer sequence was: 5’-NH_2_-(CH_2_)_6_-GCC-UGU-AAG-GUG-GUC-GGU-GUG-GCG-AGU-GUG-UUA-GGA-GAG-AUU-GC-3’ [8]. Double deionized (DI) water (18.6 MΩ) was used throughout.

### 2.2. Apparatus

The cyclic voltammetry (CV) and square wave voltammetry (SWV) studies were performed using a µStat 300 Bipotentiostat (Metrohm-Drop Sens, Llanera, Spain). The electrochemical impedance spectroscopy (EIS) studies were performed using ISX-3 impedance analyzer (Sciospec, Bennewitz, Germany). Typical EIS experiments were presented in the form of the Nyquist plot and recorded in a 16.0 mM Fe(CN)_6_^4−/3−^ solution as the redox probe. An alternating current (AC) voltage of 10 mV and a direct current (DC) voltage of 0.17 Vwereapplied over a frequency range of 100 kHz to 0.1 Hz. The EIS data were analyzed using EIS spectrum analyzer (EISSA) software. The surface morphologies of the electrodes were characterized using a scanning electron microscopy (SEM) (Field Electron and Ion (FEI, Hillsboro, OR, USA)). The elemental analysis was obtained using an energy dispersive analysis of X-rays (EDS) (EDAX, Mahwah, NJ, USA).The attenuated total reflectance spectrum (ATR) study was performed by using a Nicolet iS50 Fourier transform infrared spectrometer (Thermo Fisher Scientific, Waltham, MA, USA). 

### 2.3. Fabrication of the CSPE/CNF-CHIT/RNA Aptamer-MB

A 0.01 g amount of CNF was added to 4 mL of CHIT solution (0.5%) and the mixture was then dispersed for 1 h. After that, 5 μL CNFs-CHIT solution was dropped onto the surface of the CSPE and allowed to dry at ambient temperature. After rinsing the CSPE/CNFs-CHIT with DI water, it was immersed in a 2.5% GLU solution (phosphate-buffered saline (*PBS*), 10X, pH 7.4) for 6 h. During that process, the primary amine groups of the CHIT interacted with the aldehyde groups of GLU via the Shiff base interaction [26]. The CSPE/CNFs-CHIT-GLU electrode was then rinsed with DI water to wash away any non-bonded GLU. Subsequently, 100 µL amino-terminal of RNA aptamer solution (100 µM, 1 mM Mg^2+^, 0.1 M NaAc, pH 7.4) was dropped onto the surface of the CSPE/CNFs-CHIT-GLU to interact with the amino-terminal of RNA aptamers overnight. GLU acts as a cross-linker agent to attach the amino-terminal aptamer probes to the amino groups of the CHIT. After washing the electrode, 100 µL BSA solution (0.1 mg mL^−1^, pH 7.4) was dropped onto the electrode surface to block the non-reacted aldehyde group of GLU and avoid any non-specific binding interaction during the measurement of the CRP concentration. After that, the electrode was washed and immersed in 0.1 mM MB (10X PBS) for 1 h at ambient temperature to interact with the positively charged MB with negatively charged phosphate groups of the aptamer [21]. The CSPE/CNFs-CHIT-GLU-RNA aptamer-MB was then washed several times with PBS to wash away any loosely attached MB. The fabricated aptasensor was finally dried and stored at 4 °C when not in use.

### 2.4. Measurement Process of the CRP Concentration

**Figure 1:**
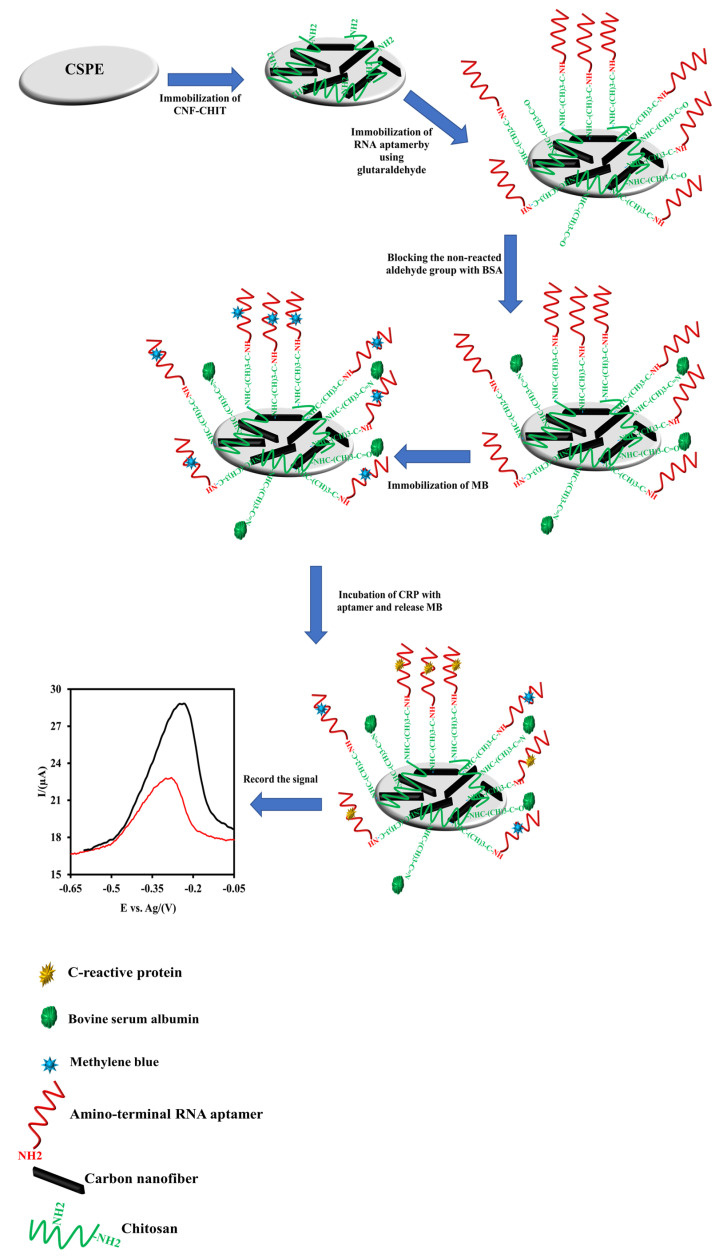
The schematic illustration for the fabrication of the CSPE/CNFs-CHIT-GLU-RNA aptamer-MB and its response mechanism to CRP.

To measure the CRP concentration, the CSPE/CNFs-CHIT-GLU-RNA aptamer-MB was immersed in different concentrations of CRP solution (0.1 M Tris buffer, pH 7.4, 2 mM CaCl_2_) to incubate the CRP with the immobilized aptamer probes. During this process, the complex of the MB-phosphate group of nucleotides will break due to the high affinity of the CRP to the RNA aptamer. In the absence of the CRP, the amount of adsorbed MB is high. Therefore, the electrochemical signal of the adsorbed MB is high. As the CSPE/CNFs-CHIT-GLU-RNA aptamer-MB is immersed in a CRP solution, the MB will release on the surface of the aptasensor to the solution and consequently, the electrochemical signal of the aptasensor will decrease. Since the amount of adsorbed MB on the aptasensor will decrease by increasing the CRP concentration, the proposed biosensor is a signal-off electrochemical aptasensor. The experimental protocol for the fabrication of the CSPE/CNFs-CHIT-GLU-RNA aptamer-MB is illustrated in Figure 1.

## 3. Results and Discussion

### 3.1. Electrochemical Activity of the Modified Electrode

Figure S1A,B show the results of the CVs of the CSPE (A) and the CSPE/CNFs-CHIT (B) in a PBS containing Fe(CN)_6_^3−/4−^ (16.0 mM (16.0 × 10^−6^ mol cm^−3^)) at different scan rates (0.01–0.15 V s^−1^). As can be seen, both the oxidation (I_pa_) and reduction peak (I_pc_) of Fe(CN)_6_^3−/4−^ increased versus the square root of the scan rate (υ^1/2^), indicating that the electrochemical kinetics are controlled by the diffusion of Fe(CN)_6_^3−/4−^ to the surface of the CSPE (Figure S1C) and the CSPE/CNFs-CHIT (Figure S1D). The electro-active area of the CSPE and the CSPE/CNFs-CHIT were also obtained by using the Randles–Ševčík equation (Equation(1)) for quasi-reversible electrochemical processes by drawing the I_p_^quasi^versus the square root of scan rate (υ^1/2^) [27]. This can use the following equation:

(1) $${I_{p}}^{\mathrm{quasi}}=\text{±}0.436\;\text{×}\;F\;\text{×}\;A{\mathrm{eas}}_{\;}\text{×}\;C\;\text{×}\;\sqrt{\frac{n\times F\times D\times \upsilon }{R\times T}}$$

where I_p_^quasi^ is the peak current, n is the number of electrons (*n* = 1), F is the Faraday constant (96,485 C mol^−1^), A_eas_ is the electroactive surface area of the electrode, C is the concentration of Fe(CN)_6_^3−/4−^ (16.0 × 10^−6^ mol cm^−3^), D is the diffusion coefficient (7.6 × 10^−6^cm^2^ s^−1^), υ is the scan rate (V s^−1^), R is the gas constant (8.314 J K^−1^ mol^−1^), and T is the temperature (298 Kelvin). The A_eas_ values for the CSPE and the CSPE/CNF-CHIT were found to be 0.14 cm^−2^ and 0.25 cm^−2^, respectively. Moreover, the roughness factor (RF) of the CSPE and the CSPE/CNFs-CHIT were found to be 1.12 and 2.0, respectively [28]. This can use the following equation:

(2) $$\mathrm{RF}=\frac{A\;\mathrm{eas}\;}{A\;\mathrm{gsa}}$$

where the geometric surface area (A_gsa_) of the CSPE is 0.125 cm^−2^. These results indicate that the CNFs-CHIT nanocomposite improved the electrochemical performances of the electrode.

Moreover, the slopes of the log I_pa_ versus log v curves for the CSPE (Figure S1E) and the CSPE/CNFs-CHIT (Figure S1F) were found to be 0.489 and 0.56 µA (Vs^−1^)^−1^, respectively, which are very close to the theoretical value of 0.5, which is specified for an ideal reaction fora diffusion-controlled process [29].

### 3.2. Surface Characterization of the Modified Electrode

**Figure 2:**
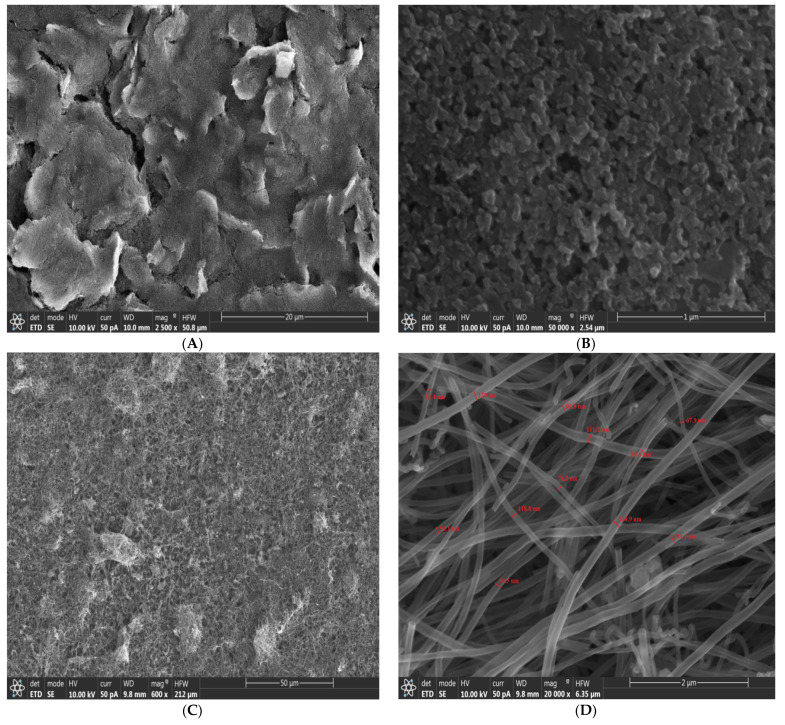
SEM images of the CSPE (**A**,**B**), CSPE/CNFs-CHIT (**C**,**D**).

The SEM images of the CSPE (A, B) and the CSPE/CNFs-CHIT (C, D) are shown in Figure 2. As shown in Figure 2A,B, the CSPE has a porous structure when compared to a glassy carbon electrode that has a smooth structure (Figure S2A,B). Moreover, Figure 2C,D show the SEM images of the CSPE/CNFs-CHIT. As can be seen, the surface of the electrode was modified with the CNFs uniformly and increased the RF of the electrode. The average diameter size of the CNFs was 102 ± 24 nm. Since CNFs have a high surface area and low electron transfer resistance, the electrochemical performance of the CSPE/CNFs-CHIT should be improved compared to the CSPE. 

The elemental analysis of the CSPE (A), the CSEP/CNFs (B), the CSPE/CNFs-CHIT (C), and the CSPE/CNFs-CHIT-GLU-RNA aptamer (D) are shown in Figure S3. It can be seen that the EDS of the CSPE/CNFs-CHIT-GLU-RNA aptamer had two new peaks related to the phosphate element and nitrogen element of the RNA aptamer (D). This proved the RNA aptamer was immobilized on the surface of the electrode. 

The ATR spectrum of the CSPE/CNFs-CHIT-GLU-RNA aptamer is also shown in Figure S4. As can be seen, a broad absorption band around 3200 cm^−1^ attributed to vibrations of the -OH group in CHIT, small peaks around 2850 cm^−1^ attributed to vibrations of the–C-H group in CLU and aptamer, a peak around 1650 cm^−1^ attributed to vibrations of the –C=N in the RNA aptamer, a peak around 1550 cm^−1^ attributed to in plan vibrations of bases in the RNA aptamer, a peak around 1240 cm^−1^ attributed to the asymmetric stretching of PO^2−^ in the phosphodiester groups of the RNA aptamer, and a peak around 1080 cm^−1^ attributed to the symmetrical stretching of -PO^2−^ in the phosphodiester groups of the RNA aptamer [30,31].

### 3.3. Electrochemical Characterization of the Assembled Interface of the Surface of the RNA Aptasensor

**Figure 3:**
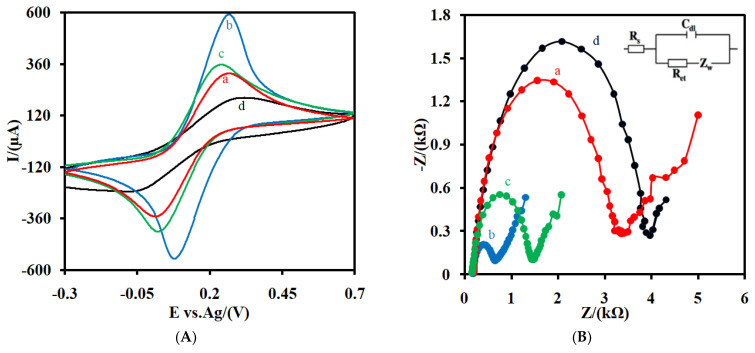

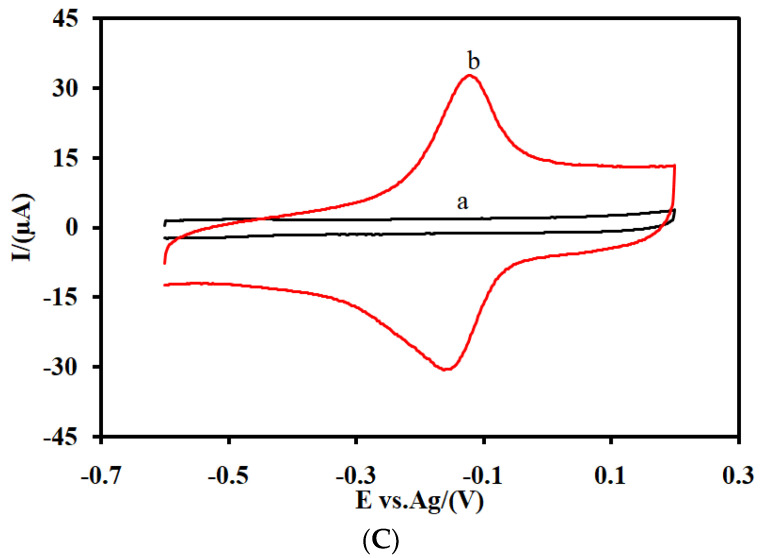
(**A**) CVs and EIS (**B**) of CSPE (a) CSPE/CNFs-CHIT (b), CSPE/CNFs-CHIT-GLU-RNA aptamer (c), and CNFs-CHIT-GLU-RNA aptamer/CRP (d) in 0.1 M 16.0 mM Fe(CN)_6_^3−/4−^ solution (0.1 M PBS, pH 7.4) at a scan rate of 0.05 Vs^−1^. (**C**) CVs of the CSPE/CNFs-CHIT-GLU-RNA aptamer (a), the CSPE/CNFs-CHIT-GLU-RNA aptamer-MB (b) at a PBS at a scan rate of 0.05 Vs^−1^. The inset of Figure 3B is the equivalent electric circuit compatible with the Nyquist diagrams. *R*_s_: Solution resistance; *R*_et_: Electron transfer resistance; *C*_dl_: Double-layer capacitance; *Z*_w_: Warburg impedance. AC amplitude was 10 mV, DC potential was 0.17 V, and frequency range was 0.1 Hz–100 kHz.

CV (Figure 3A) and EIS (Figure 3B) were also used as efficient methods for studying the interface properties of the CSPE (a), the CSPE/CNFs-CHIT (b), and the CSPE/CNFs-CHIT-GLU-RNA aptamer (c) in the presence of the Fe(CN)_6_^3−/4−^ redox couple. As shown in Figure 3A, after the modification of the CSPE (a) with CNFs-CHIT (b), the intensity of the peak currents not only increased but also the difference between the potential of the anodic peak and the potential of the cathodic peak (ΔE = E_pa_ − E_pc_) decreased from 0.25 V for the CSPE (a) to 0.18 V for the CSPE/CNFs-CHIT (b). This indicated that the CNFs-CHIT nanocomposite facilitated the electron transfer rate of the Fe(CN)_6_^3−/4−^ redox couple. After the immobilization of the aptamer on the surface of the CSPE/CNFs-CHIT, the intensity of the peak current decreased (c). Moreover, the ΔE of the CV increased from 0.18 V for the CSPE/CNFs-CHIT to 0.22 V for the CSPE/CNFs-CHIT-GLU-RNA aptamer. The reasonable explanation for this is that the electrostatic repulsion interaction between the immobilized aptamer on the surface of the electrode and Fe(CN)_6_^3−/4−^ limited the accessibility of Fe(CN)_6_^3−/4−^ to the surface of the electrode. As CRP (20.0 pM) was incubated with the RNA aptamer on the surface of the CSPE/CNFs-CHIT-GLU-RNA aptamer, the intensity of the peaks decreased and ΔE increased (0.41 V). The reasonable explanation for this is that the incubated CRP on the surface of the RNA-based aptasensor limited the mass-transfer diffusion of Fe(CN)_6_^3−/4−^ to the surface of the RNA-based aptasensor.

The stepwise change in the electrochemical behavior of the electrodes was also studied using the EIS method (Figure 3B). As can be seen, after the modification of the CSPE with CNFs-CHIT, the electron transfer resistance (R_et_) decreased from 3047 Ω for the CSPE (a) to 490 Ω for the CSPE/CNFs-CHIT (b). The R_et_ increased to 1253 Ω as the RNA aptamers were attached to the surface of the electrode by GLU (c). Finally, the R_et_ increased to 3674 Ω after the incubation of CRP with the attached RNA aptamer on the surface of the electrode (d). The results were consistent with the CV results. This change in the CV and EIS response of the aptasensor before (c) and after the incubation with CRP (d) indicated that the proposed RNA aptasensor is sensitive to this biomarker. 

Figure 3C shows the CVs for the CSPE/CNFs-CHIT-GLU-RNA aptamer (a) and the CSPE/CNFs-CHIT-GLU-RNA aptamer-MB (b) at PBS. As can be seen, no redox peak was observed for the CSPE/CNFs-CHIT-GLU-RNA aptamer. However, a couple of well-defined and reversible redox peaks were observed for the adsorbed MB on the aptasensor, indicating that the MB was incubated with the aptamer.

CVs of a CSPE/CNFs-CHIT-GLU-RNA aptamer-MB were recorded for different scan rates (ʋ) at PBS. (Figure S5A). As shown, the log I_pa_ of MB has a linear relationship with logʋ (Figure S5B). The slope of the log I_pa_ versus logʋ curve for the CSPE/CNFs-CHIT-GLU-RNA aptamer-MB was found to be 1.08 µA V^−1^ s^−1^, very close to the theoretical value of 1.0, which is specified for an ideal adsorption-controlled process [29].

The surface coverage of MB (Γ_MB_) was estimated from the integration of the anodic peak in the cyclic voltammogram of the CSPE/CNFs-CHIT-GLU-RNA aptamer-MB (Figure S6) [32]. This is according to Equation (3):

(3) $$\Gamma_{\mathrm{MB}}\;=\frac{Q\;\mathrm{Faradic}}{\mathrm{nFA}}$$

where Q_Faradic_ is the integrated area of the oxidation peak (peak area/scan rate; the scan rate was 0.05) (Coulomb), n is the number of electrons (*n* = 2), F is the Faraday constant (9648 C mol^−1^), and A is the electroactive surface area of the electrode (0.25 cm^−2^). The value of Γ_MB_ was calculated to be 0.99 nmol cm^−2^. The theoretical surface coverage (Γ_Theory_) of the monolayer absorbance of MB at the surface of the electrode was0.22 (nmol cm^−2^) [33]. Therefore, the ratio of Γ_MB_ to Γ_Theory_ (η = Γ/Γ_Theory_) was calculated to be 4.5 for the proposed aptasensor. This value is much larger than the theoretical value. The reasonable explanation for this is that the CNFs-CHIT nanocomposite increased the surface roughness of the electrode. 

Figure S6A shows how the Faradic charge (Q_Faradic_) was obtained for the anodic peak current of the CSPE/CNFs-CHIT-GLU-RNA aptamer-MB. As shown, the total charge Q_Total_ includes the non-Faradic and Faradic charge:

Q_Total_ = Q_Faradic_ + Q_Non-Faradic_ (4)

After the subtraction of non-Faradic charge (Q_Non-Faradic_) from Q_Total_ (the charge below the red area), the Q_Faradic_ can be obtained (Figure S6B). The value of Q_Faradic_ for the CSPE/CNFs-CHIT-GLU-RNA aptamer-MB was 47.7 × 10^−6^ coulomb.

The aptamer surface coverage (T_Aptamer_) on the modified electrode was also investigated. Since every CRP aptamer probe sequence contains 44 phosphodiester groups and a phosphodiester group interacts with an MB, the Γ_MB_ can be converted to Γ_Aptamer_ [34]. This is done by using Tarlov Equations (5) and (6):

(5) $$\Gamma_{\mathrm{Aptamer}}\;=\Gamma_{\mathrm{MB}}\frac{z}{m}$$

(6) $$\Gamma_{\mathrm{Aptamer}}\;=\Gamma_{\mathrm{MB}}\frac{z}{m}\times N_{A}$$

where z is the charge of the adsorbed molecule (z = 1 for MB), m is the number of phosphate groups of aptamer (m = 44), and N_A_ is Avogadro’s number (6.022 × 10^23^ molecules mol^−1^). 

The value of Γ_Aptamer_ was calculated to be 22.5 pmol cm^−2^ (by using Equation (5)) or 1.35 × 10^13^ molecules cm^−2^ (by using Equation (6)),which was greater than previously reported for the CRP aptamer probe [8]. The value of Γ_Aptamer_ on the CSPE/CHIT-GLU-RNA aptamer-MB was also obtained with the same method used for the CSPE/CNFs-CHIT-GLU-RNA aptamer-MB (Figure S7A–C). The values of Q_Faradic_, T_MB_, and Γ_Aptamer_ for the CSPE/CHIT-GLU-RNA aptamer-MB were obtained to be 4.8 × 10^−6^ coulomb, 0.17 nmol cm^−2^, and 3.8 pmol cm^−2^ (2.32 × 10^+12^ molecules cm^−2^), respectively. 

Figure S8A,B show the CVs and the anodic peak current related to the Faradic charge of MB on the CSPE/CNFs-CHIT-GLU-RNA aptamer-MB (a), and the CSPE/CHIT-GLU-RNA aptamer-MB (b). As can be seen, the Faradic charge of MB on the CSPE/CNFs-CHIT-GLU-RNA aptamer-MB was much bigger than on the CSPE/CHIT-GLU-RNA aptamer-MB. The obtained result indicated that the CNFs-CHIT nanocomposite increased the Γ_Aptamer_ value up to 5.9 times.

The heterogeneous electron transfer rate constant (K_s_) for MB was also calculated for the CSPE/CNFs-CHIT-GLU-RNA aptamer-MB using Laviron’s formula for the surface controlled electrochemical system (ΔEp < 200 mV, α = 0.5) [35]. This is shown in Equation (7):

(7) $$K_{s}=\frac{m\times n\times F\times \upsilon }{R\times T}$$

where m is the parameter related to the peak potential separation, n the number of electrons involved in the reaction, ν is the scan rate (V s^−1^), F is the Faraday constant (96,485 C mol^−1^), R is the universal gas constant (8.31 J K^−1^ mol^−1^), and T is the temperature (298 Kelvin). The average value of K_s_ for MB was found to be 1.83 s^−1^, suggesting that the electron transfer of the self-assembled MB on the proposed aptasensor has good reversibility.

### 3.4. Optimization of the Response Time and the RNA Aptamer Probe Concentration on the Response of the CSPE/CNFs-CHIT-GLU-RNA Aptamer-MB

**Figure 4:**
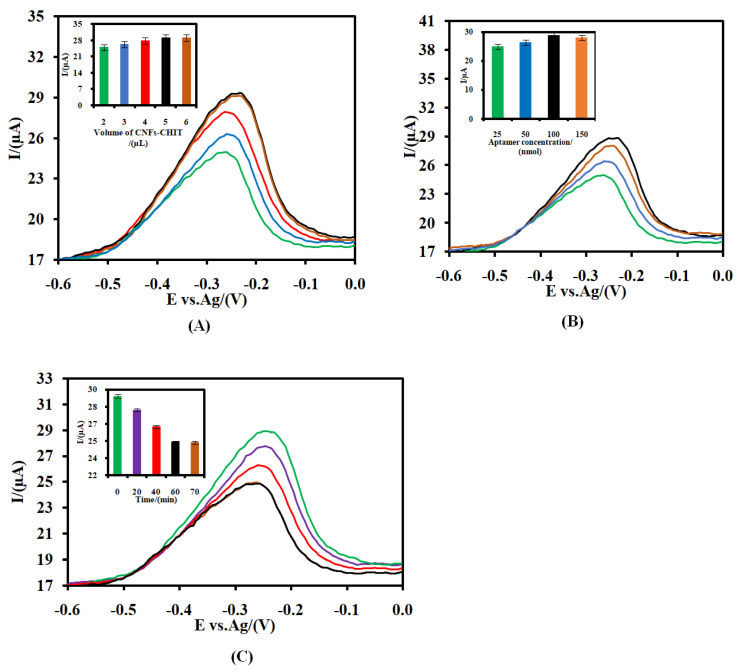
(**A**) Effect of CNFs-CHIT volume, (**B**) the concentration of RNA aptamer on the response of CSPE/CNFs-CHIT-GLU-RNA aptamer-MB. (**C**) The effect of the incubation time to detect 20 pM CRP at a PBS. The error bars were obtained by using four different aptasensors.

The influence of the volume of the CNFs-CHIT (A), the concentration of the immobilized RNA aptamer on the amount of the self-assembled MB (B), and the incubation time on the response of the CSPE/CNFs-CHIT-GLU-RNA aptamer-MB to 20.0 pM CRP (C) were investigated (Figure 4) by using the one-factor-at-a-time method. 

As can be seen in Figure 4A, the signal of the assembled MB on the surface of the RNA aptasensor increased by increasing the volume of the CNFs-CHIT from 2 to 5 µL and then decreased. The effect of the RNA aptamer probe on the signal of the aptasensor was also investigated (Figure 4B). As shown, the signal increased by increasing the concentration of the RNA aptamer solution from 25.0 nmol to 100.0 nmol and then decreased as the concentration of the RNA aptamer solution increased to 150.0 nmol. This is due to the electrostatic repulsion between the negatively charged aptamer strands’ increase in the high concentration of RNA aptamer, hindering them in their interaction with GLU. Therefore, the amount of the immobilized RNA aptamer and consequently the amount of the immobilized MB decreased [36].

The effect of the incubation time was also studied (Figure 4C). As can be seen, the signal of the self-assembled MB on the aptasensor in the presence of 20.0 pM CRP decreased from 20 min to 60 min, and after that remained unchanged, suggesting that the bond between MB and the phosphate groups of the aptamer broke and a new bond between CRP and the aptamer (CRP/RNA aptamer) was generated. The results indicated that the interaction of the RNA aptamer with the CRP reached a saturation level after 60 min. Therefore, we chose 5 µL CNFs-CHIT, 100 nmol RNA aptamer to fabricate the RNA aptasensor, and 60 min as the incubation time between the CRP and RNA aptamer as the optimized conditions throughout this work.

### 3.5. Voltammetric Detection of CRP

**Figure 5:**
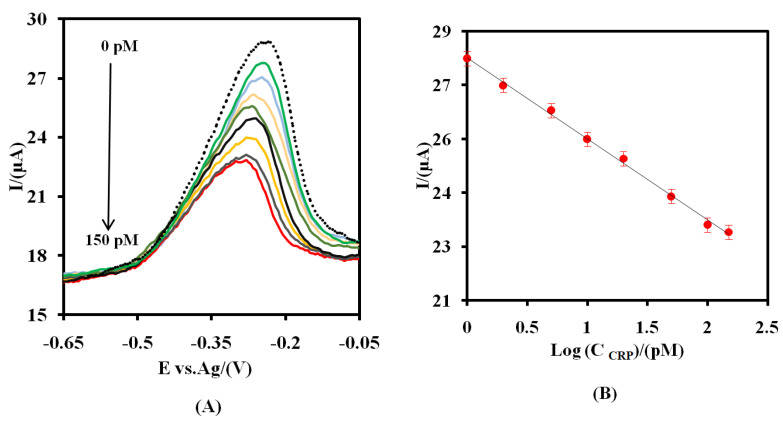

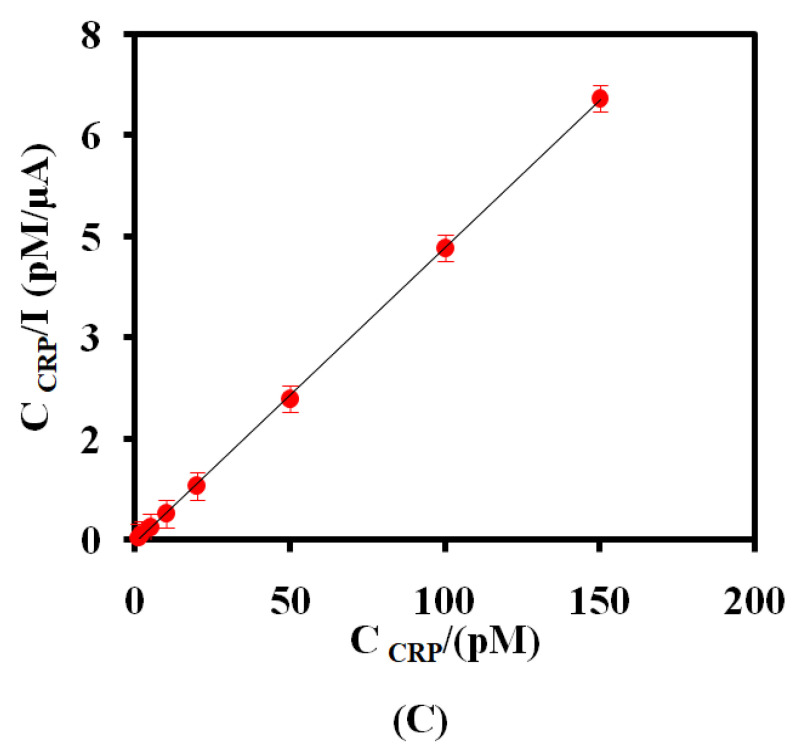
(**A**) The SWV of the CSPE/CNFs-CHIT-GLU-RNA aptamer-MB at the optimum operating conditions for different amounts of CRP (1.0, 2.0, 5.0, 10.0, 20.0, 50.0, 100.0, and 150.0 pM) at a PBS. (**B**) The logarithmic calibration curve plot. (**C**) The plat of Langmuir binding isotherm model. The error bars were obtained by using four different aptasensors.

The SWV method was applied for the measurement of the various concentrations of CRP (Figure 5A). As shown, the signal of the proposed aptasensor to the different concentrations of CRP (C_CRP_) decreased logarithmically as the C_CRP_ increased from 1.0 to 150.0 pM (Figure 5B). The corresponding logarithmic regression equation was:

I_p_ (μA) = −2.26 Log C_CRP_ (pM) + 27.77 (8)

The sensitivity was 9.0 μA pM^−1^ cm^−2^. The limit of detection (LOD) was found to be 0.37 pM (3σ/S), where σ is the standard deviation of the blank measurements for four different aptasensors (signal of the aptasensor in the absence of CRP) and S the slope of Figure 5B. The error bars represent the calculated standard deviation for the four different aptasensors. As can be seen, the intensity of the SWV signals is not only decreased but also the position of the signal was shifted. To explain this change in the peak position, the CVs of the CSPE/CNF-CHIT-GLU-RNA aptamer-MB in the absence and presence of 50.0 pM CRP were recorded (Figure S9). As shown, the intensity of the signal not only decreased but also the ΔE in the CV of MB increased from 0.052 V (in the absence of CRP) to 0.163 V. The reasonable explanation is that as a big molecule like CRP is incubated with the RNA aptamer, the electron transfer to the surface of the electrode for the rest of the assembled MB would be difficult, and consequently, the ΔE in the CV of MB increased.

The value of the Langmuir isotherm constant (K_L_), the dissociation constant (K_d_), and the maximum number of binding sites (I_max_) for the CRP-RNA aptamer were also obtained using the Langmuir adsorption systems equation [37]. Equation (9) is as follows:

(9) $$\frac{C\;\mathrm{CRP}}{I}=\frac{1}{\mathrm{KL}\times \mathrm{Imax}}+\frac{C\;\mathrm{CRP}}{\mathrm{Imax}}$$

where I is the steady-state current after the addition of the biomarker.

The values of 1/I_max_ and 1/K_L_ × I_max_ can be obtained from the slope (0.043) and intercept point (−0.04) in Figure 5C, respectively. The values of I_max_, K_L_, and K_d_ (1/K_L_) were found to be 23.25 µA, 1.07 pM^−1^, and 0.93 pM, respectively. The value of K_d_ is lower than the previously reported RNA aptasensor for CRP [8], indicating the high affinity of the proposed aptasensor. This indicates that the high surface area of the modifier (CNFs-CHIT) prepared the better position and orientation conditions for the bio-recognizer (RNA aptamer) to interact with its target (CRP).

Moreover, the Gibbs free energy for the desorption band of the CRP-RNA aptamer was found to be −179.6 kJ mol^−1^ [38]. This is done by using the equation below:

ΔG = 2.03 × R × T × log (K_d_) (10)

where R is the universal gas constant of 8.31 J K^−1^ mol^−1^, and T is the temperature (298.15 Kelvin).

The analytical performance of the CSPE/CNFs-CHIT-GLU-RNA aptamer-MB was compared with other aptasensors for CRP measurement (Table 1). It is obvious that the CSPE/CNFs-CHIT-GLU-RNA aptamer-MB has good analytical performance in comparison with other aptasensors for CRP due to using the CNFs-CHIT nanocomposite.

### 3.6. Stability, Reproducibility, and Selectivity of the CSPE/CNFs-CHIT-GLU-RNA Aptamer-MB

The selectivity of the CSPE/CNFs-CHIT-GLU-RNA aptamer-MB was also studied (Figure S10). As can be seen, no interference was detected for 10-fold quantities of HSA and HIgG in the determination of 10.0 pM CRP, indicating the proposed aptasensor has good selectivity. Figure S11 shows the stability of the CSPE/CNFs-CHIT-GLU-RNA aptamer-MB after two weeks. As shown, no significant change in the signal is observed (~2.4%), indicating the high stability of the proposed RNA aptasensor. The high stability of the CSPE/CNFs-CHIT-GLU-RNA aptamer-MB can be attributed to the biocompatible microenvironment provided by the CNFs-CHIT.

The reproducibility of the CSPE/CNFs-CHIT-GLU-RNA aptamer-MB was evaluated by three newly fabricated aptasensors (Figure S12). The relative standard deviation (RSD) was 3.1 % for 10.0 pM CRP.

### 3.7. Analytical Application of the Modified Electrode

Human serum samples were prepared based on the previous report. Details are included in the supplementary data section. Serum samples 40.0 μL in amount were added to 156.0 µL PBS and mixed for 15 min. Then the solution was divided into two samples. After that, 2 µL CRP solution with different concentrations (100 pM, and 500 pM) was added to each of the two solutions and mixed for 15 min. The CRP concentration was 2.0 pM for the first diluted serum sample and 10.0 pM for the second one. The CRP concentration in the real sample was calculated by using the calibration curve (Figure 5B). Figure S13 shows the responses of the aptasensor to sample 1 (2.0 pM) and sample 2 (10.0 pM). The obtained results were compared with a CRP-ELISA kit (Table S1). As can be seen, the P-values were greater than 0.05. Therefore, there were no statistically significant differences between the responses of the proposed RNA aptasensor and a CRP-ELISA kit. 

While the CSPE/CNFs-CHIT-GLU-RNA aptamer-MB demonstrated excellent analytical performance in terms of sensitivity, stability, and selectivity, its response time is slower than the immunosensors. Therefore, our future plans include designing a new aptamer probe to decrease the response time of the sensor.

## 4. Conclusions

In this study, an electrochemical RNA aptasensor was introduced with a strategy based on the association of the CNFs, CHIT, and MB to measure CRP concentration. The investigations indicated that the CNFs-CHIT nanocomposite increased the aptamer surface coverage dramatically. The proposed RNA aptasensor for the CRP measurement had a wider linear-response range and a lower LOD, and an affinity. The obtained results also demonstrated that the proposed RNA aptasensor has good stability, reproducibility, and selectivity. Moreover, there were not any significant differences between the responses of the proposed RNA aptasensor and a CRP-ELISA kit in human serum samples. The analytical performances of the proposed RNA aptasensor were not only better than the aptasensors previously reported for CRP, but also better than the immunosensors previously reported for CRP (Table S2). This is because of the low electron transfer resistance, the high surface area, and the high number of the primary amine functional groups of the CNFs-CHIT nanocomposite. We hope that the proposed aptasensor paves the way for a new opportunity to fabricate a cost-effective, selective, and sensitive device to measure the CRP biomarker as one of the key biomarkers in sepsis diseases.

## Figures and Tables

**Table 1:** Comparison of the analytical performance of the CSPE/CNFs-CHIT-GLU-RNA Aptamer-MB with other reported aptasensors for CRP.

Aptasensor	Detection Technique	Linear Response Range	LOD	K_d_	Г_aptamer_	Ref.
MBs-Aptamer-AuNP	Dark-field microscopy	6.1–49 nM	2.71 nM	-	-	[5]
Gold elecrode/RNA aptamer-MB	SWV	1–100 pM	1 pM	25.9 pM	4.98 pmol cm^−2^	[8]
Gold elecrode/DNA aptamer-MB	SWV	1–100 pM	1 pM	6 pM	8.3 pmol cm^−2^	[25]
Gold-streptavidin-Biotinated aptamer	SPR	0.005–0.5 ppm	0.005 ppm	125 nM	-	[14]
GCE/AuNP/Aptamer-and SiO_2_-APTES-AuNP-Antibody/BSA-Zn^2+^ ion	SWV	5.0–125.0 pg mL^−1^	7.0 pg mL^−1^	-	-	[39]
CSPE/CNFs-CHIT-RNA aptamer-MB	SWV	1–150 pM	0.37 pM	0.93 pM	53.8 pmol cm^−2^	This work

MBs: Magnetic beads; AuNP: Gold nanoparticles; GCE: Glassy carbon electrode; APTES: 3-Aminopropyltriethoxysilane.

## Supplementary Materials

The following supporting information can be downloaded at: https://www.mdpi.com/article/10.3390/nano12030415/s1, Figure S1: CVs of the CSPE (A) and CSPE/CNF-CHIT (B) in 16.0 mM Fe(CN)63−/4− solution (0.1 M PBS, pH 7.4) at various scan rates (0.01. 0.025, 0.05, 0.075, 0.1, 0.125, and 0.15from inner to outer). The plot of the anodic peak current (Ipa) and cathodic peak current (Ipc) versus square root of scan rate (ν) for CSPE (C) and CSPE/CNF-CHIT (D). The plot of the logarithm of the anodic peak current (Ipa) versus the logarithm of scan rate (ν) for CSPE (E) and CSPE/CNF-CHIT (F). Figure S2: SEM images (A,B) of a glassy carbon electrode. Figure S3: EDS of CSPE (A), CSPE/CNFs (B), CSPE/CNFs-CHIT (C), CSPE/CNFs-CHIT-GLU-RNA aptamer (D). Figure S4: ATR spectrum of the CSPE/CNFs-CHIT-GLU-RNA aptamer. Figure S5: CVs of the CSPE/CNFs-CHIT-GLU-RNA aptamer-MB (A) in a PBS at various scan rates (0.01. 0.025, 0.05, 0.075, 0.1, 0.125, 0.15, 0.175, 0.2, 0.225, and 0.25 Vs-1 from inner to outer). The plot of the logarithm of the anodic peak current (Ipa) versus the logarithm of scan rate (ν) for CSPE/CNF-CHIT-GLU-RNA aptamer-MB (B). Figure S6: The anodic peak current obtained from CVof the CSPE/CNFs-CHIT-GLU-RNA aptamer-MB includes non-Faradic current (a area) and Faradic current (b area) (A) at a scan rate of 0.05 Vs−1. The anodic peak current (Faradic current) was obtained from the CV of the CSPE/CNF-CHIT-GLU-RNA aptamer-MB after the subtraction of non-Faradic current from the total current (B). Figure S7: CV of the CSPE/CHIT-GLU-RNA aptamer-MB (A) in a PBS at a scan rate of 0.05 V.s−1. The anodic peak current obtained from CVof CSPE/CNFs-CHIT-GLU-RNA aptamer-MB includes non-Faradic current and Faradic current (B). The anodic peak current (faradic current) obtained from CVof the CSPE/CNFs-CHIT-GLU-RNA aptamer-MB after the subtraction of non-Faradic current from the total current (C). Figure S8: CVs (A) and Faradic anodic current (B) of the CSPE/CNFs-CHIT-GLU-RNA aptamer-MB (a) and the CSPE/CHIT-GLU-RNA aptamer-MB (b) in a PBS at a scan rate of 0.05 Vs-1. Figure S9: CVs of the CSPE/CNFs-CHIT-GLU-RNA aptamer-MB in a PBS in the absence (a) and presence of 50 pM CRP (b) at a scan rate of 0.05 Vs-1. Figure S10: SWVs of the CSPE/CNFs-CHIT-GLU-RNA aptamer-MB to 10 pM CRP in a PBS (0.1 M, pH 7.4) in the absence (a) and presence of 100 pM HSA and 100 pM HIgG (b). Figure S11: SWVs of the CSPE/CNFs-CHIT-GLU-RNA aptamer-MB in a PBS (0.1 M, pH 7.4) in the first day (a) and 2 weeks after its fabrication (b). Figure S12: SWVs of the three different aptasensors (a–c) to 10 pM CRP in a PBS related to the reproducibility of the aptasensor. Figure S13: SWVs of the CSPE/CNFs-CHIT-GLU-RNA aptamer-MB in the 5-fold diluted plasma serum sample with PBS in the absence (a) and presence of 2 pM (b) and 10 pM CRP (c). Table S1: Comparison of the obtained results between the proposed RNA aptasensor and an ELISA kit. Table S2: Comparison of the analytical performance of the CSPE/CNF-CHIT-GLU-RNA Aptamer-MB with the other immunosensors for CRP.
