# Supplementary material for: Highly Sensitive RNA-Based Electrochemical Aptasensor for the Determination of C-Reactive Protein Using Carbon Nanofiber-Chitosan Modified Screen-Printed Electrode
Source: Nanomaterials (Basel). 2022 Jan 27;12(3):415. doi: 10.3390/nano12030415 (PMC8839947; doi:10.3390/nano12030415)
Supplement: Supplementary file 1 [file nanomaterials-12-00415-s001.zip › nanomaterials-1523016-SI.pdf]

nanomaterials

MDPI

Supplementary Materials

Highly Sensitive RNA-Based Electrochemical Aptasensor for the Determination of C-Reactive Protein Using Carbon Nanofiber-Chitosan Modified Screen-Printed Electrode

Mahmoud Amouzadeh Tabrizi * and Pablo Acedo *

Electronic Technology Department, Universidad Carlos III de Madrid, 28911 Leganés, Spain
* Correspondence: mamouzad@ing.uc3m mahmoud.tabrizi@gmail.com (M.A.T.); pag@ing.uc3m.es (P.A.)

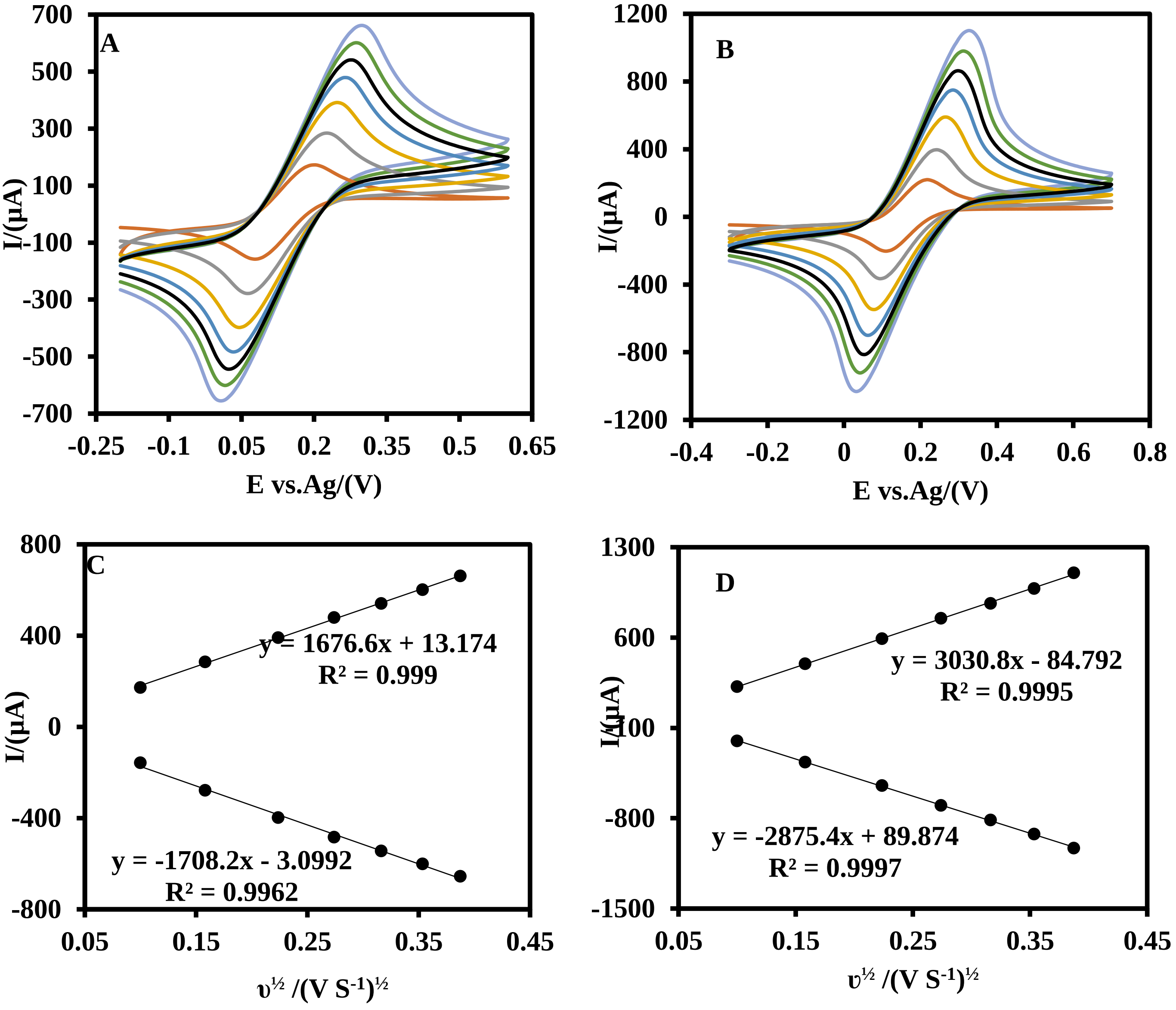

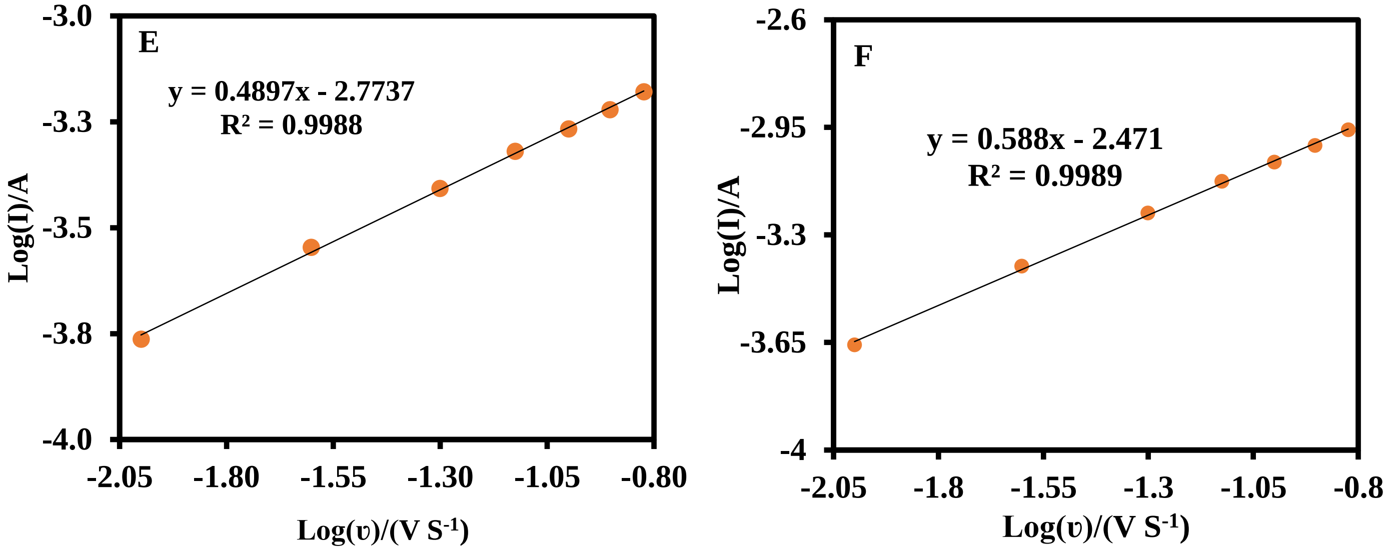

Figure S1. CVs of the CSPE (A) and CSPE/CNF-CHIT (B) in 16.0 mM $Fe(CN)_6^{3-/4-}$ solution (0.1M PBS, pH 7.4) at various scan rates (0.01. 0.025, 0.05, 0.075, 0.1, 0.125, and 0.15from inner to outer). The plot of the anodic peak current (I_{pa}) and cathodic peak current (I_{pc}) versus square root of scan rate (ν) for CSPE (C) and CSPE/CNF-CHIT (D). The plot of the logarithm of the anodic peak current (I_{pa}) versus the logarithm of scan rate (ν) for CSPE (E) and CSPE/CNF-CHIT (F).

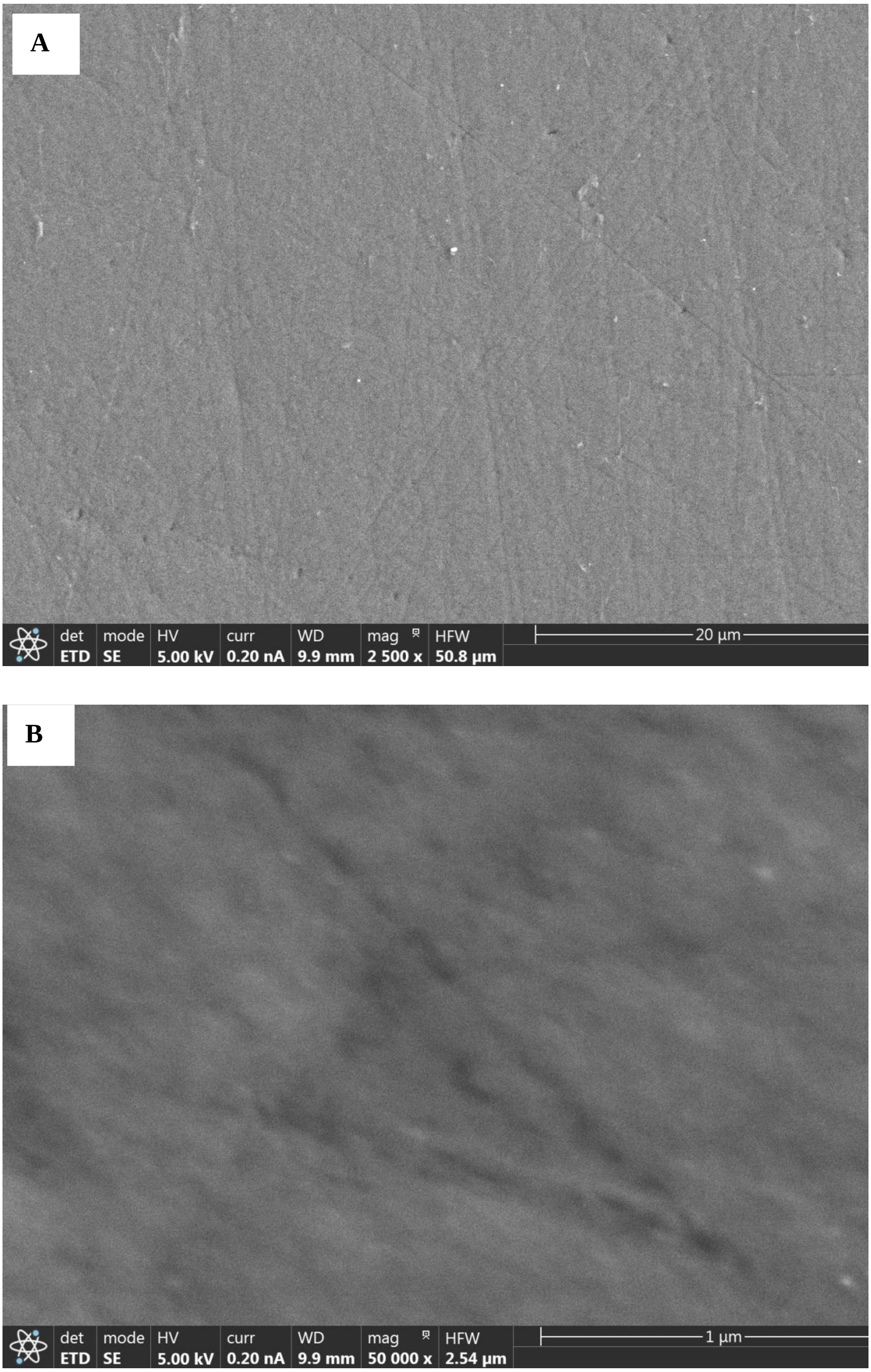

Figure S2. SEM images (A, B) of a glassy carbon electrode.

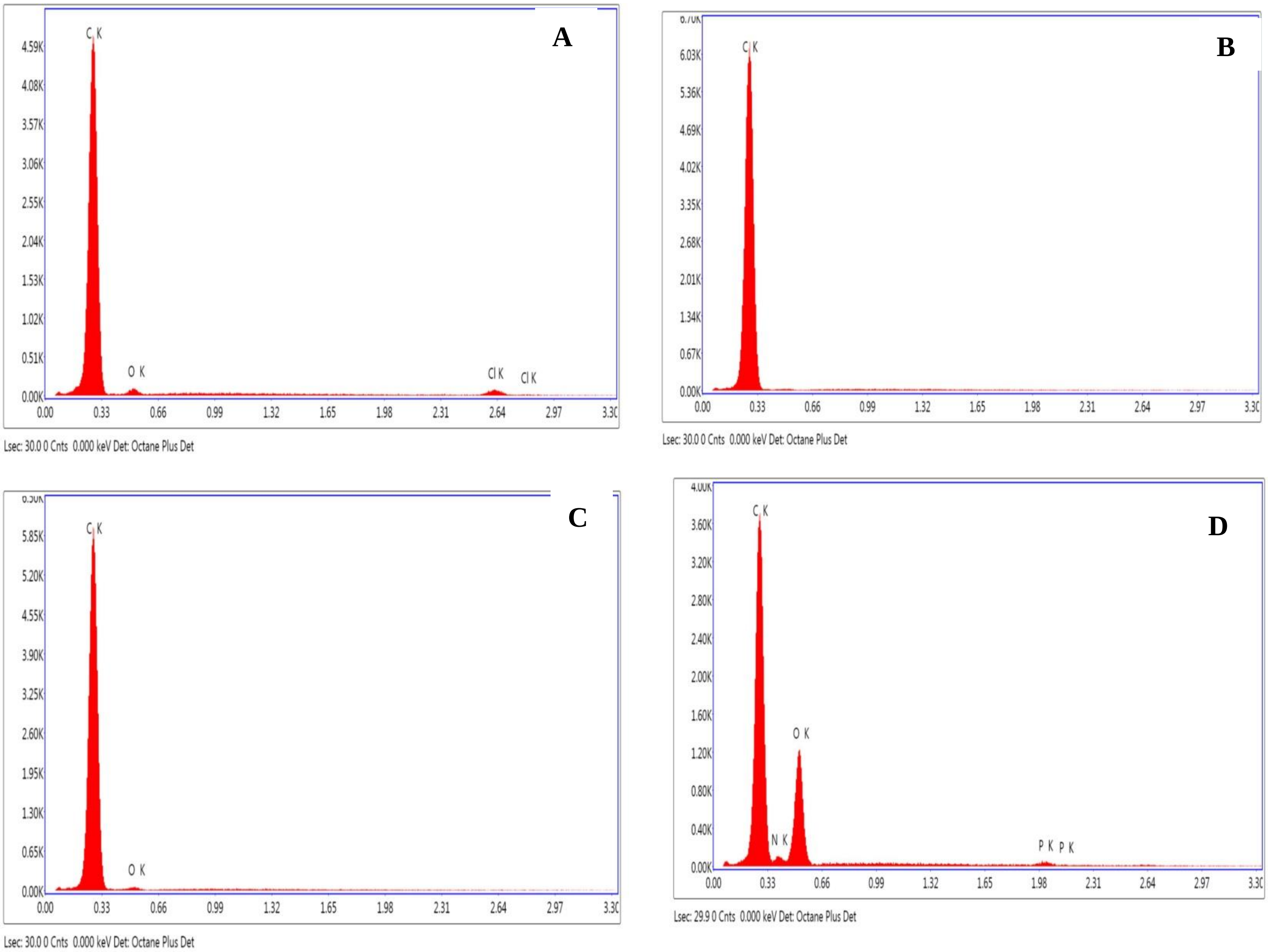

Figure S3. EDS of CSPE (A), CSPE/CNFs (B), CSPE/CNFs-CHIT (C), CSPE/CNFs-CHIT-GLU-RNA aptamer (D).

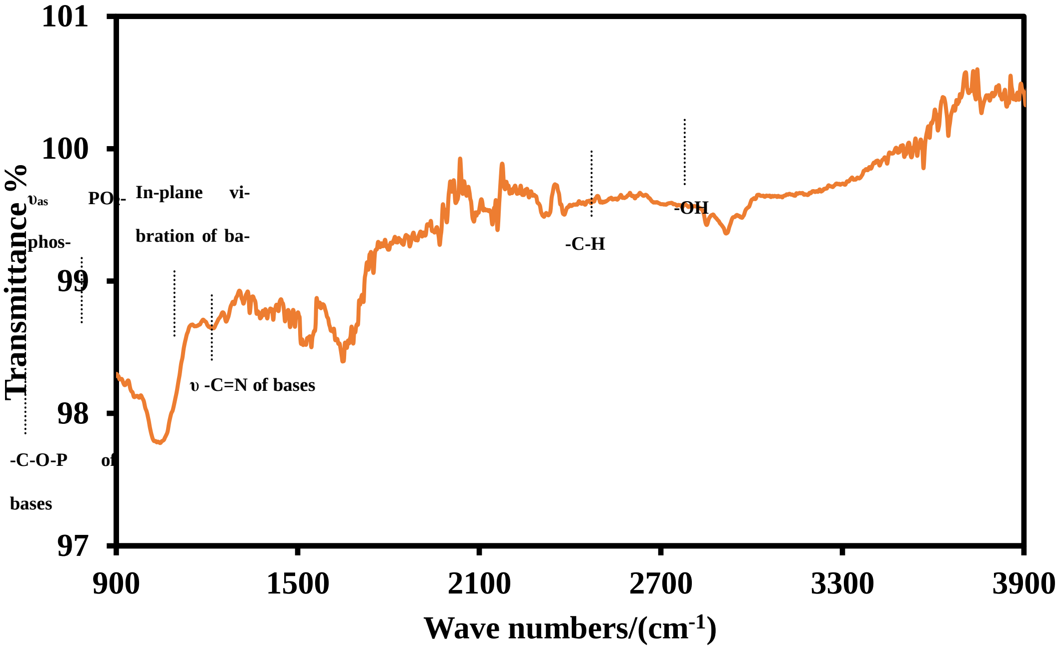

Figure S4. ATR spectrum of the CSPE/CNFs-CHIT-GLU-RNA aptamer.

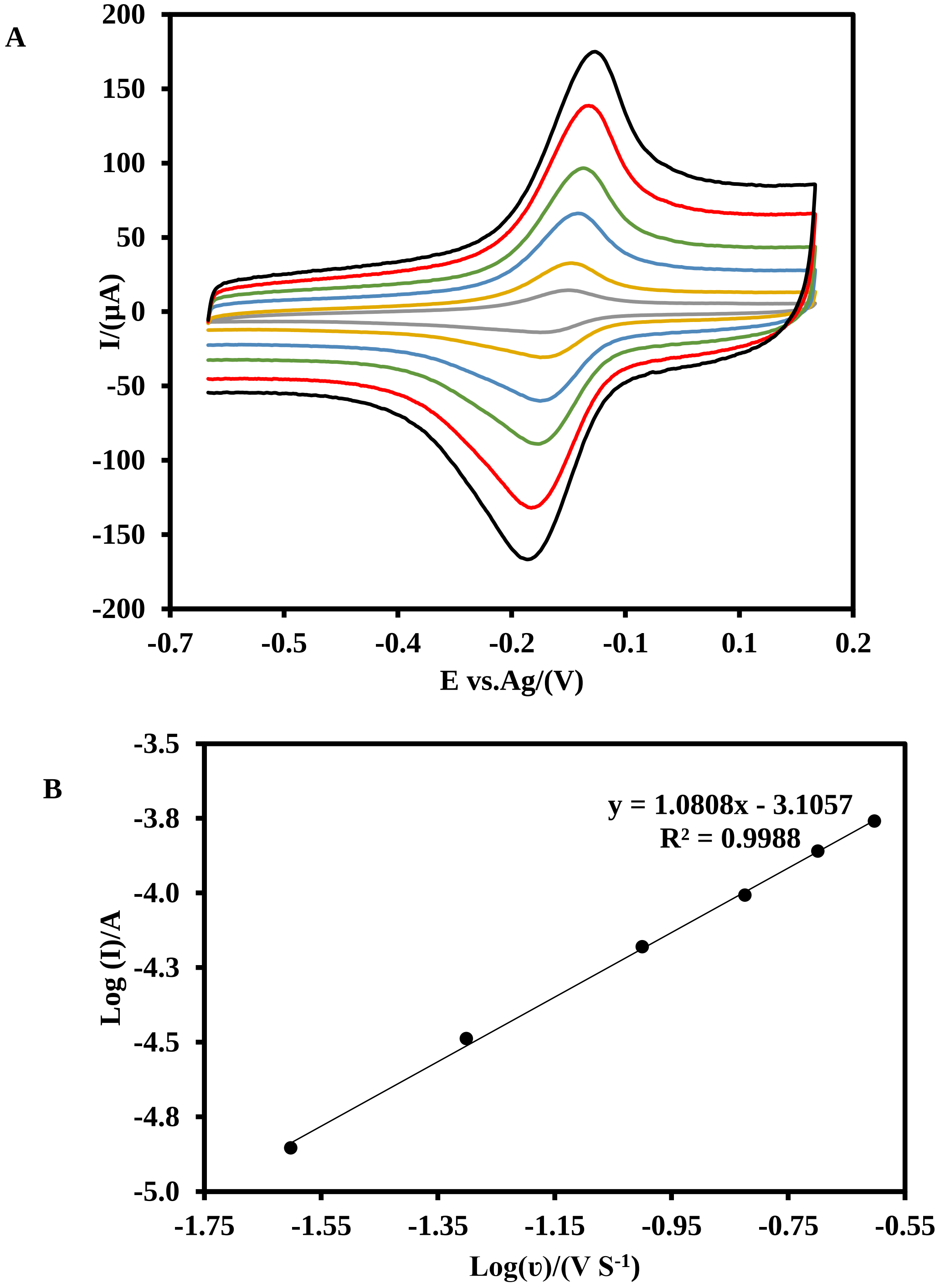

Figure S5. CVs of the CSPE/CNFs-CHIT-GLU-RNA aptamer-MB (A) in a PBS at various scan rates (0.01. 0.025, 0.05, 0.075, 0.1, 0.125, 0.15, 0.175, 0.2, 0.225, and 0.25 Vs^{-1} from inner to outer). The plot of the logarithm of the anodic peak current (I_{pa}) versus the logarithm of scan rate (ν) for CSPE/CNF-CHIT-GLU-RNA aptamer-MB (B).

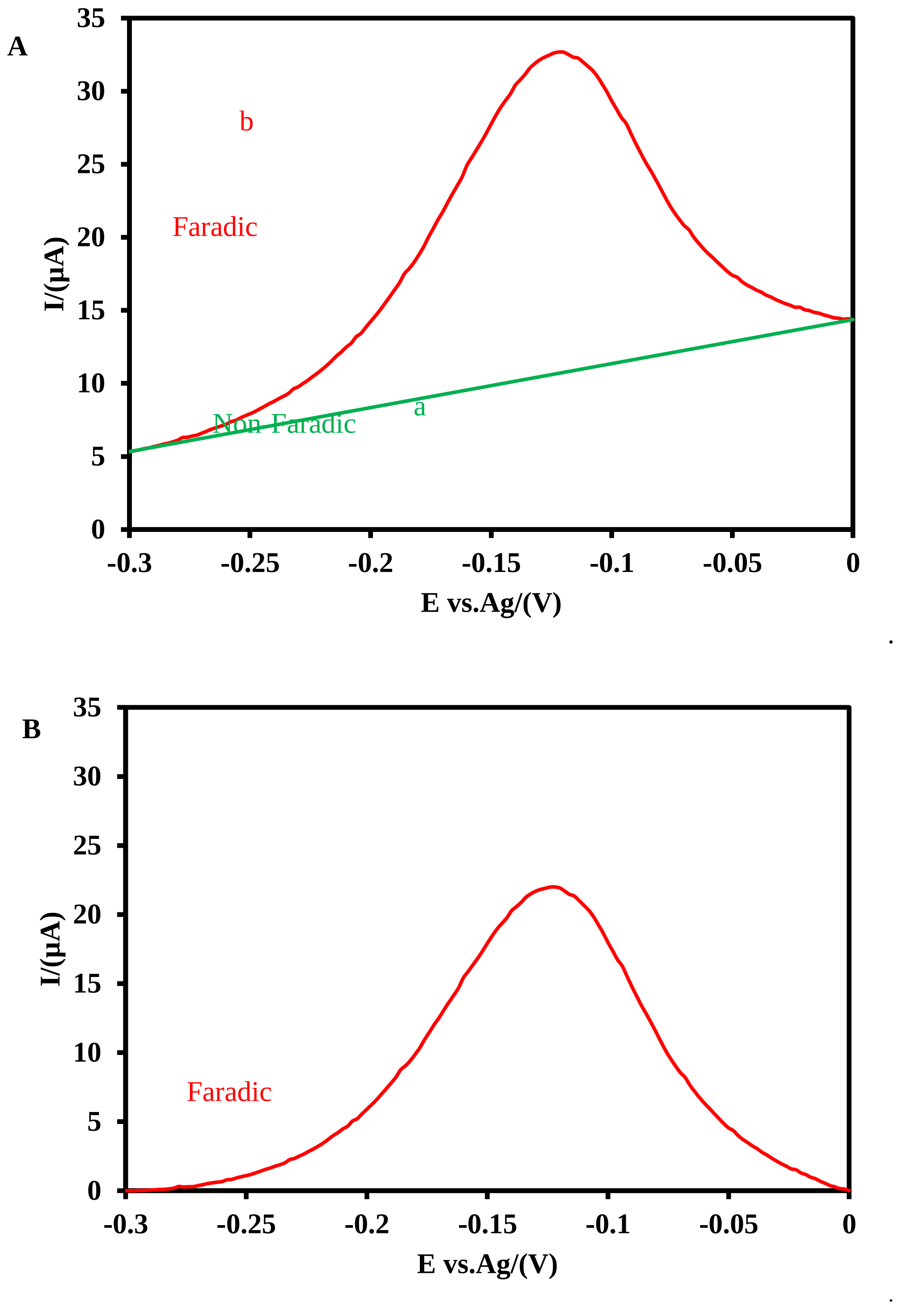

Figure S6. The anodic peak current obtained from CVof the CSPE/CNFs-CHIT-GLU-RNA aptamer-MB includes non-Faradic current (a area) and Faradic current (b area) (A) at a scan rate of 0.05 Vs^{-1}. The anodic peak current (Faradic current) was obtained from the CV of the CSPE/CNF-CHIT-GLU-RNA aptamer-MB after the subtraction of non-Faradic current from the total current (B).

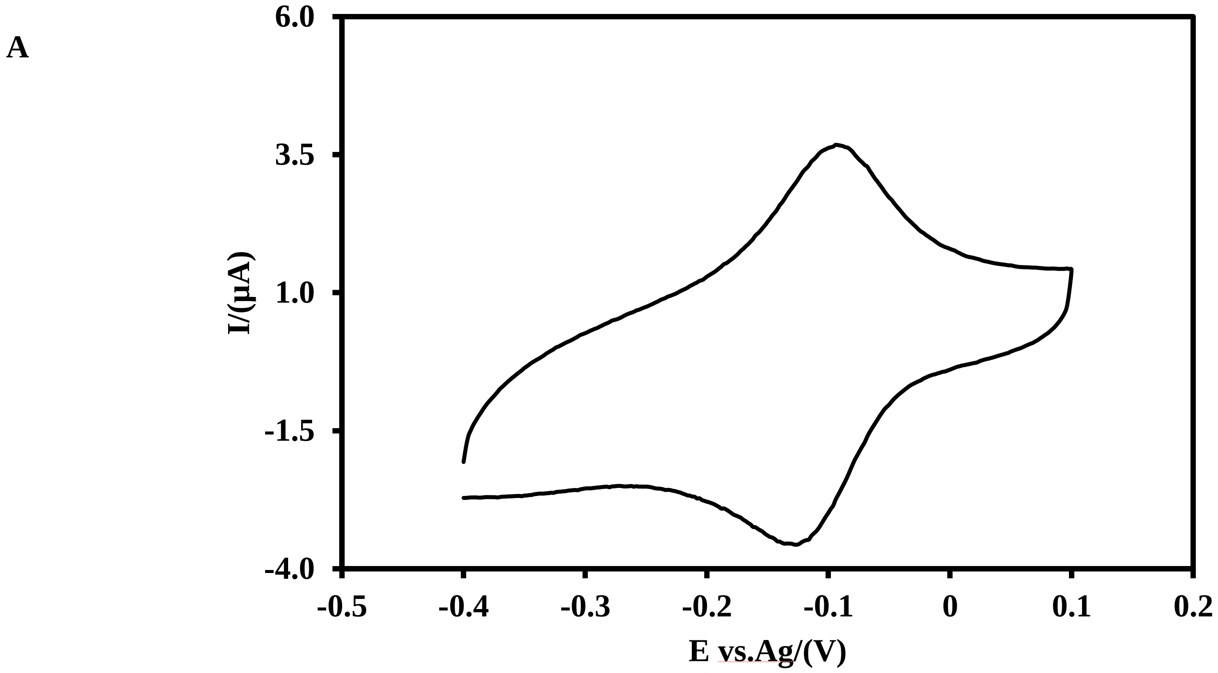
A
6.0
3.5
1.0
-1.5
-4.0
I/(μA)
-0.5
-0.4
-0.3
-0.2
-0.1
0
0.1
0.2
E vs.Ag/(V)

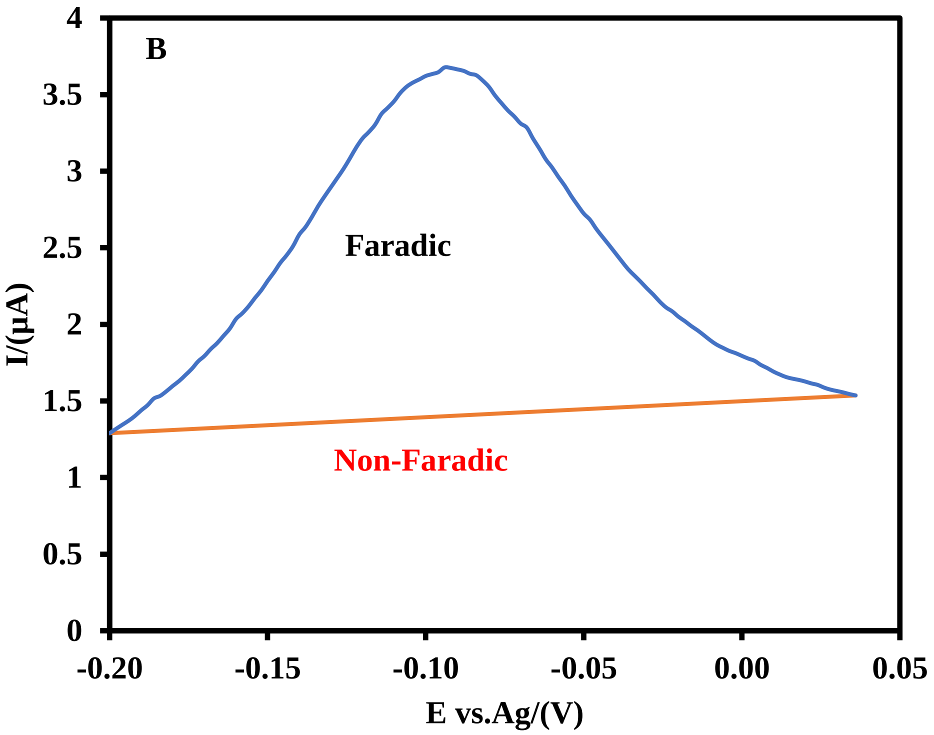
B
4
3.5
3
2.5
2
1.5
1
0.5
0
I/(μA)
Faradic
Non-Faradic
-0.20
-0.15
-0.10
-0.05
0.00
0.05
E vs.Ag/(V)

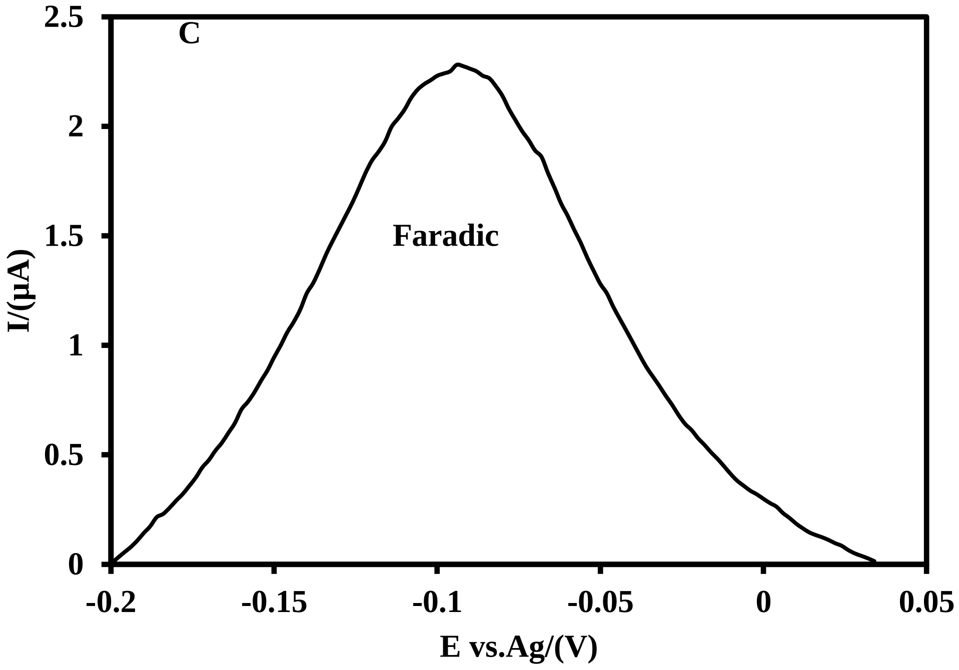

Figure S7. CV of the CSPE/CHIT-GLU-RNA aptamer-MB (A) in a PBS at a scan rate of 0.05 V.s^{-1}. The anodic peak current obtained from CVof CSPE/CNFs-CHIT-GLU-RNA aptamer-MB includes non-Faradic current and Faradic current (B). The anodic peak current (faradic current) obtained from CVof the CSPE/CNFs-CHIT-GLU-RNA aptamer-MB after the subtraction of non-Faradic current from the total current (C).

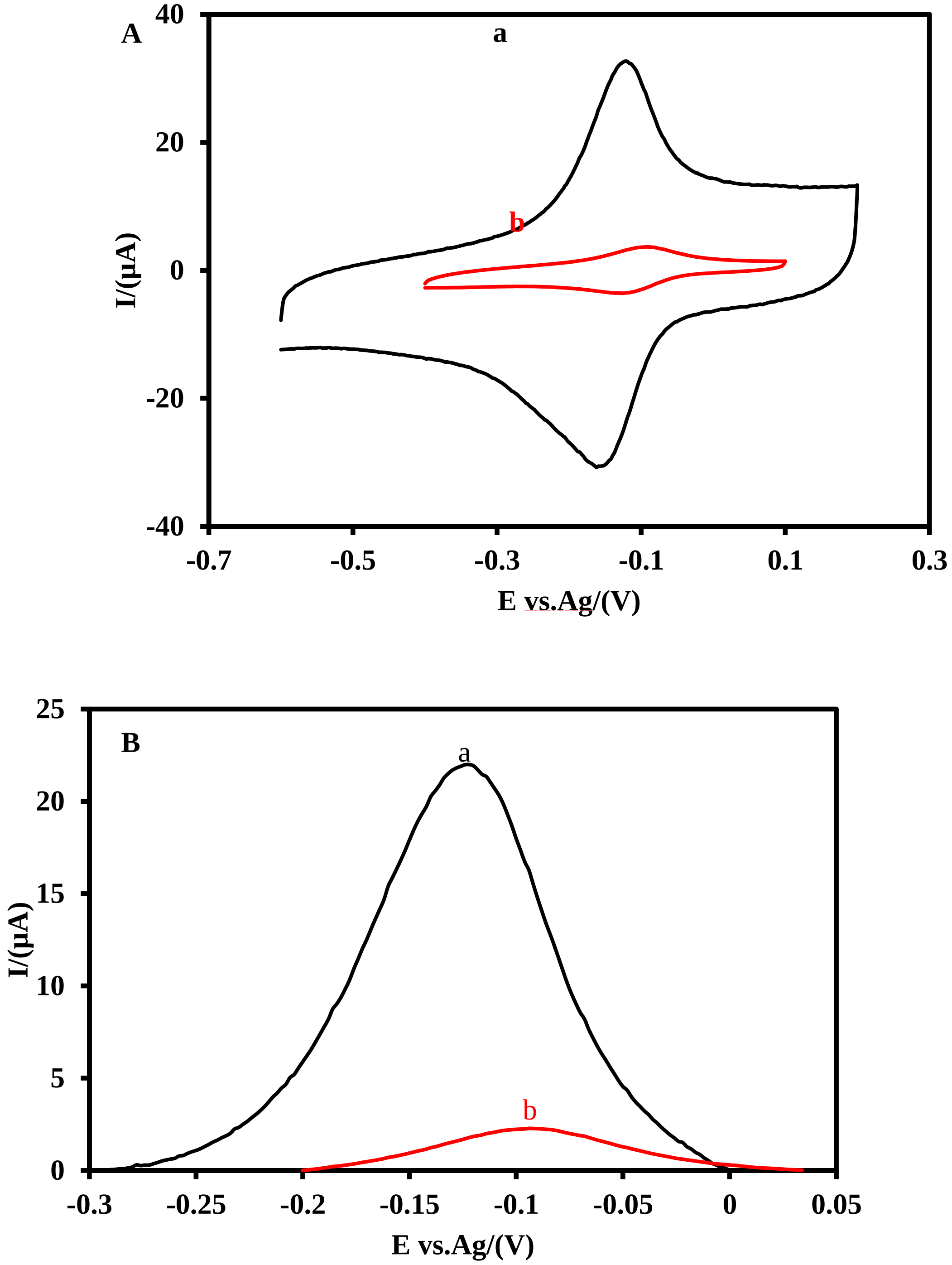

Figure S8. CVs (A) and Faradic anodic current (B) of the CSPE/CNFs-CHIT-GLU-RNA aptamer-MB (a) and the CSPE/CHIT-GLU-RNA aptamer-MB (b) in a PBS at a scan rate of 0.05 Vs^{-1}.

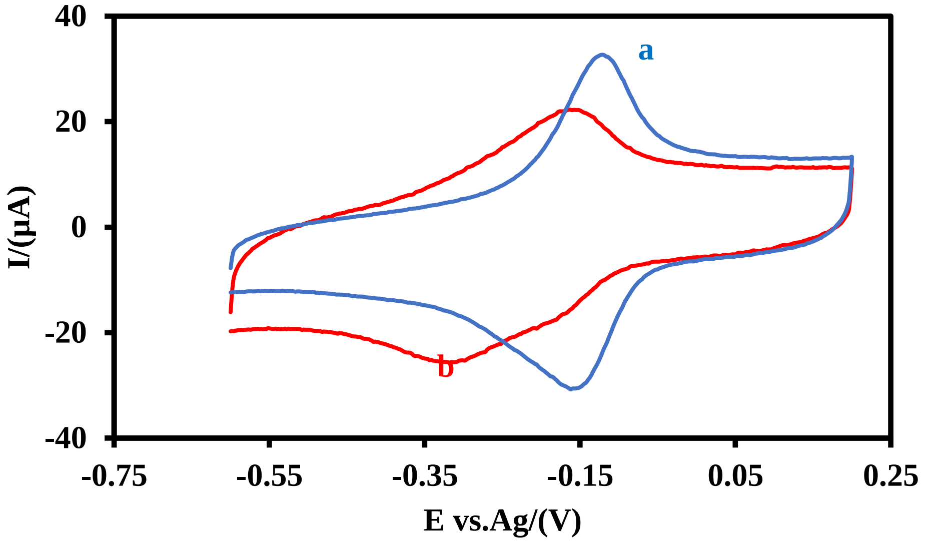

Figure S9. CVs of the CSPE/CNFs-CHIT-GLU-RNA aptamer-MB in a PBS in the absence (a) and presence of 50 pM CRP (b) at a scan rate of 0.05 Vs^{-1}.

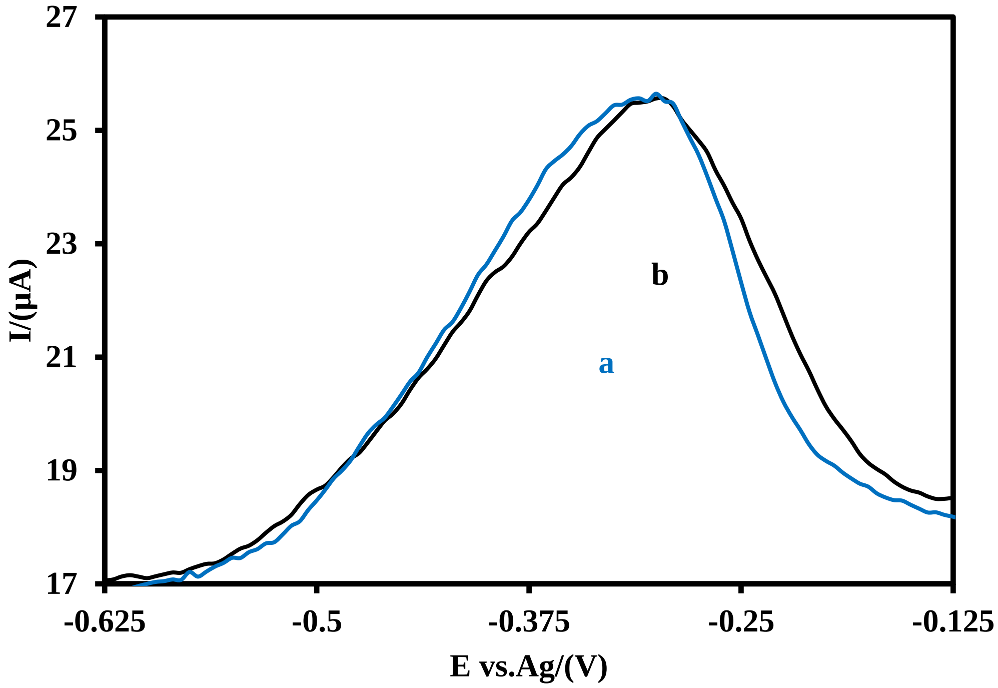

Figure S10. SWVs of the CSPE/CNFs-CHIT-GLU-RNA aptamer-MB to 10 pM CRP in a PBS (0.1 M, pH 7.4) in the absence (a) and presence of 100 pM HSA and 100 pM HIgG (b).

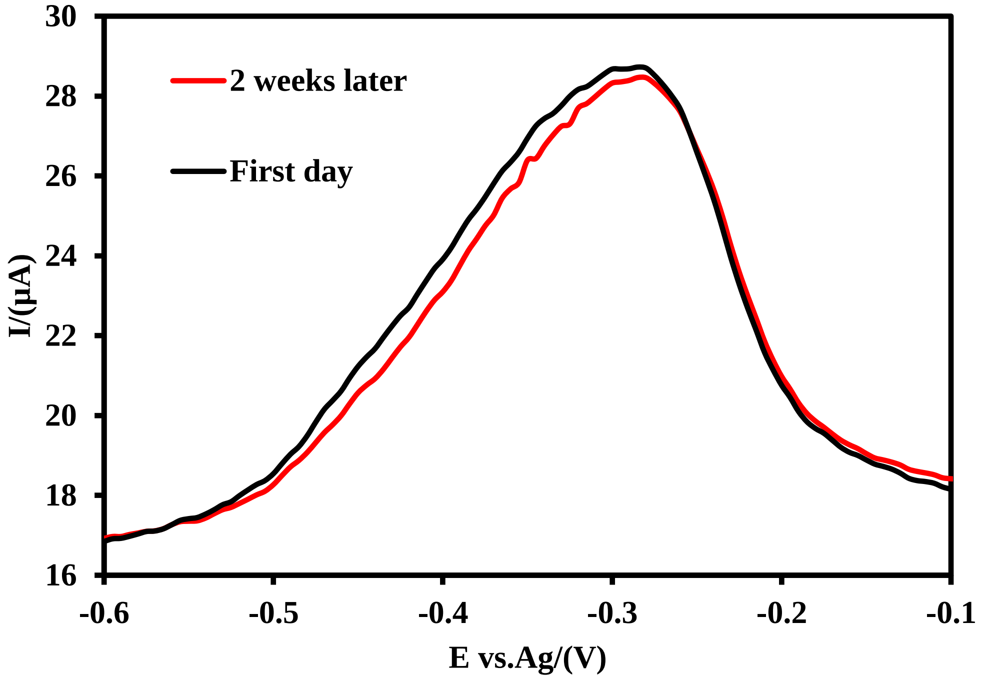

Figure S11. SWVs of the CSPE/CNFs-CHIT-GLU-RNA aptamer-MB in a PBS (0.1 M, pH 7.4) in the first day (a) and 2 weeks after its fabrication (b).

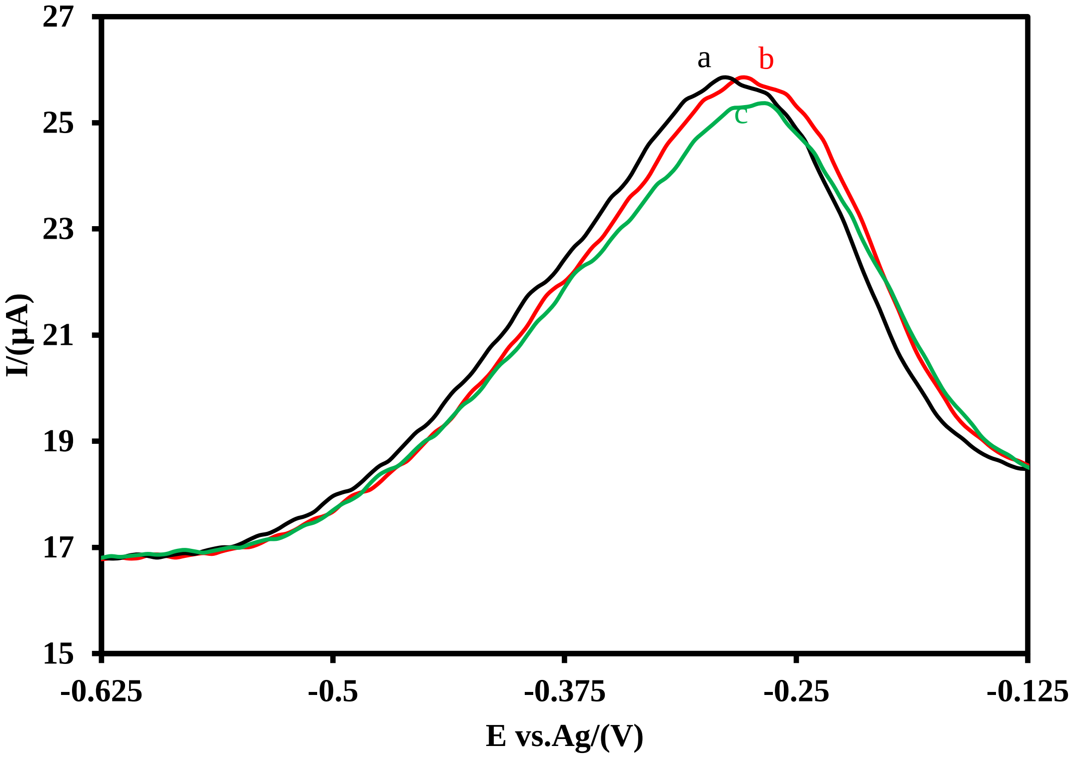

Figure S12. SWVs of the three different aptasensors (a-c) to 10 pM CRP in a PBS related to the reproducibility of the aptasensor.

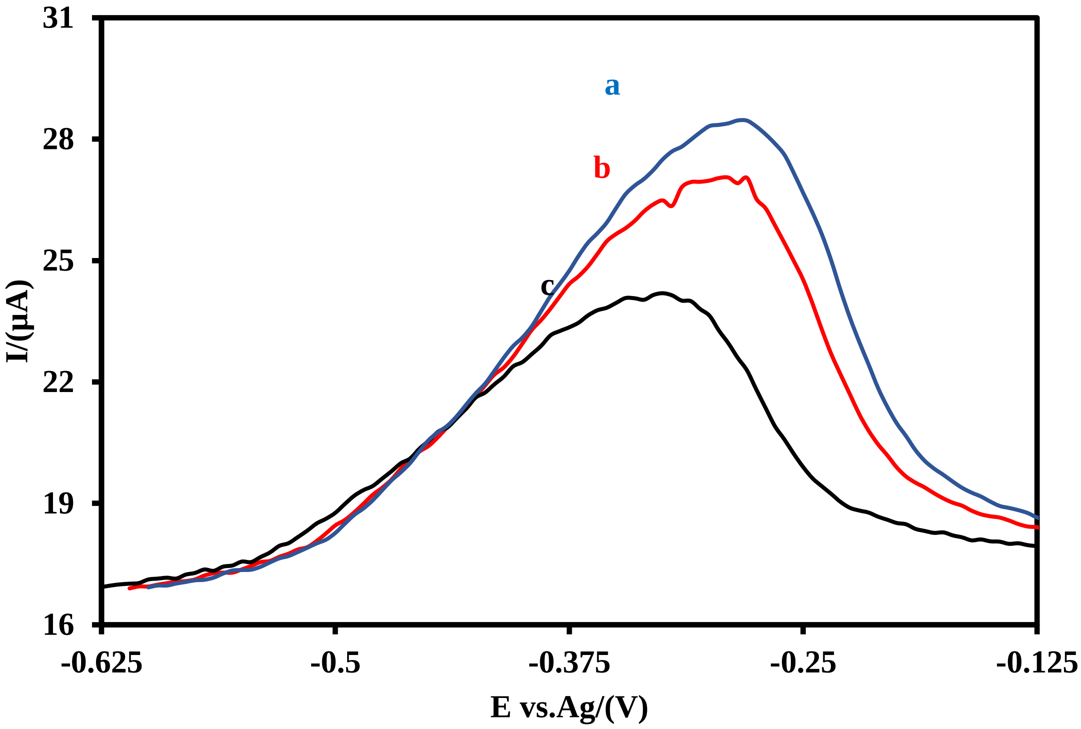

Figure S13. SWVs of the CSPE/CNFs-CHIT-GLU-RNA aptamer-MB in the 5-fold diluted plasma serum sample with PBS in the absence (a) and presence of 2 pM (b) and 10 pM CRP (c).

Preparation of human serum sample

5 mL of fresh human blood was transferred into a tube and kept at room temperature for 20 min. Then, the human blood was centrifuged for 10 min at 3000 rpm. Finally, a part of the above solution was separated and divided into small tubes. The prepared human serum samples were then stored at −80 °C until use.

Table S1. Comparison of the obtained results between the proposed RNA aptasensor and an ELISA kit.

Sample	Obtained concentration by MIP sensor/(pM)	Mean/(pM)	Standard deviation	Count	Standard error of mean	Degree of freedom	Hypothesized mean/(pM)	T-value	P-value	Obtained concentration by ELISA kit/(pM)
1	2.0; 1.9, 2.1; 2.2	2.05	0.13	4.0	0.064	3.0	2.0	0.77	0.49	2.0
2	10.3; 10.1; 9.75; 10.0	10.03	0.22	4.0	0.11	3.0	10.0	0.32	0.74	10.0

Table S2. Comparison of the analytical performance of the CSPE/CNF-CHIT-GLU-RNA Aptamer-MB with the other immunosensors for CRP.

Biosensor	Detection technique	Linear range	LOD	Ref
Gold/ZnO/succinimidyl propionate/Anti-CRP	EIS	0.01-20 µg mL^{-1}	0.1 µg mL^{-1}	[1]
C-SPE/Au_{nano}/Cysteine Anti-CRP	CAM	0.047–23.6 µg/mL	0.15 nM	[2]
Au nanowire/ 3-mercaptopropionic acid/Anti-CRP	SWV	5–220 fg mL^{-1}	2.25 fg mL^{-1}	[3]
Gold/11-mercaptoundecanoic acid and 3,3-dithiodipropionic acid/ Anti-CRP	CAM	2.2 to 100 ng mL^{-1}	2.2 ng mL^{-1}	[4]
SPE/Aunano/Thiol-terminated poly(2-methacryloyloxyethyl phosphorylcholine)/Ca^{2+}	DPV	5- 5000 ng mL^{-1}	1.6 ng/mL^{-1}	[5]
Giant magnetoimpedance/ Anti-CRP and functionalized magnitic bead with anti-CRP as a secondary antibody	EIS	1-10 ng mL^{-1}	1 ng mL^{-1}	[6]
Gold/11-mercaptoundecanoic acid/ Anti-CRP	EGOTFT	210-6×10^{14} zM	210 zM	[7]
ZnO/Polyethylene terephthalate/ Anti-CRP	EIS	1-15 ng.mL^{-1}	1 ng mL^{-1}	[8]
ITO/Titania nanotubes/ Platinum nanowire/Anti-CRP	ECL	0.05-6.25 ng	0.011 ng	[9]
GCE/ graphene quantum dots/ PEG-thiol/Anti-CRP	EIS	0.5–70 nM	176 pM	[10]
ITO/3-cyanopropyltrimethoxysilane/ Anti-CRP	EIS	3.25–208 fg. mL^{-1}	0.455 fg mL^{-1}	[11]
CSPE/CNF-CHIT-RNA aptamer-MB	SWV	1-150 pM	0.37 pM	The work

CAM: Chronoamperometry; DPV: Differential pulse voltammetry; EGOTFT: Electrolyte-gated organic thin-film transistor

References

1. Tanak, A.S.; Jagannath, B.; Tamrakar, Y.; Muthukumar, S.; Prasad, S. Non-faradaic electrochemical impedimetric profiling of procalcitonin and c-reactive protein as a dual marker biosensor for early sepsis detection. *Anal. Chim. Acta X* **2019**, *3*, 100029.
2. Thangamuthu, M.; Santschi, C.; O, J.F.M. Label-free electrochemical immunoassay for c-reactive protein. *Biosensors* **2018**, *8*.
3. Vilian, A.T.E.; Kim, W.; Park, B.; Oh, S.Y.; Kim, T.; Huh, Y.S.; Hwangbo, C.K.; Han, Y.-K. Efficient electron-mediated electrochemical biosensor of gold wire for the rapid detection of c-reactive protein: A predictive strategy for heart failure. *Biosens. Bioelectron.* **2019**, *142*, 111549.
4. Fakanya, W.M.; Tothill, I.E. Detection of the inflammation biomarker c-reactive protein in serum samples: Towards an optimal biosensor formula. *Biosensors* **2014**, *4*, 340-357.

5. Pinyorospathum, C.; Chaiyo, S.; Sae-ung, P.; Hoven, V.P.; Damsongsang, P.; Siangproh, W.; Chailapakul, O. Disposable paper-based electrochemical sensor using thiol-terminated poly(2-methacryloyloxyethyl phosphorylcholine) for the label-free detection of c-reactive protein. Mikrochim *Acta* **2019**, *186*, 472.
6. Yang, Z.; Liu, Y.; Lei, C.; Sun, X.-c.; Zhou, Y. A flexible giant magnetoimpedance-based biosensor for the determination of the biomarker c-reactive protein. Mikrochim *Acta* **2015**, *182*, 2411-2417.
7. Macchia, E.; Manoli, K.; Holzer, B.; Di Franco, C.; Picca, R.A.; Cioffi, N.; Scamarcio, G.; Palazzo, G.; Torsi, L. Selective single-molecule analytical detection of c-reactive protein in saliva with an organic transistor. *Anal. Bioanal. Chem.* **2019**, *411*, 4899-4908.
8. Cao, L.; Kiely, J.; Piano, M.; Luxton, R. Facile and inexpensive fabrication of zinc oxide based bio-surfaces for c-reactive protein detection. *Sci. Rep.* **2018**, *8*, 12687.
9. Rong, Z.; Chen, F.; Jilin, Y.; Yifeng, T. A c-reactive protein immunosensor based on platinum nanowire / titania nanotube composite sensitized electrochemiluminescence. *Talanta* **2019**, *205*, 120135.
10. Bing, X.; Wang, G. Label free c-reactive protein detection based on an electrochemical sensor for clinical application. *Int. J. Electrochem. Sci.* **2017**, *12*, 6304 – 6314.
11. Sonuç Karaboğa, M.N.; Sezgintürk, M.K. A novel silanization agent based single used biosensing system: Detection of c-reactive protein as a potential alzheimer's disease blood biomarker. *J. Pharm. Biomed. Anal.* **2018**, *154*, 227-235.
